# Influence of the Post-Processing on the Surface Quality and the Mechanical Properties of Alumina Parts Processed by Ceramic Material Extrusion Additive Manufacturing

**DOI:** 10.3390/ma19050998

**Published:** 2026-03-05

**Authors:** Thomas Heim, Frank Kern

**Affiliations:** Institute for Ceramic Materials and Technologies (IKMT), University of Stuttgart, Allmandring 7B, 70569 Stuttgart, Germany; frank.kern@ikmt.uni-stuttgart.de

**Keywords:** fused deposition of ceramic, alumina fused filament fabrication, surface roughness, four point bending strength, dip-coating, aerosol surface treatment

## Abstract

This paper presents an evaluation of two new approaches to improve the surface quality and the mechanical properties of ceramic parts printed by fused deposition of ceramic (FDC). Dip-coating and aerosol-treatment are performed in order to reduce the staircase effect in the vertical printing direction, which typically represents the weakest orientation in most additive manufacturing processes, particularly in fused filament fabrication (FFF). The post-treatments are applied on two highly filled alumina feedstocks. A commercial aerosol-treatment machine for fused deposition modeling is used with ethanol as solvent. A suspension composition for dip-coating is developed to reduce the surface roughness without compromising the printing resolution. The influence of these post-processing steps on the mechanical properties and surface roughness of the green and sintered parts is investigated using perthometer measurements and four-point bending tests in the vertical build direction on as-processed, aerosol-treated, and dip-coated samples. The mechanical results are compared to extruded strand samples. An improvement in surface quality is achievable by dip-coating despite reduction in the parts strength, with a reduction of 65% of the R_z_ values in the sintered state compared to untreated samples. Aerosol-treatment neither improves the surface quality nor the mechanical properties of the parts. The feedstock and post-processing steps developed in this research aim at printing dense ceramic parts with high surface quality, serving as a basis for developing ceramic parts with higher strength. This advancement will facilitate the utilization of FDC in structural and aesthetic design applications.

## 1. Introduction

The implementation of additive manufacturing in the ceramics industry is progressing much more slowly than in the polymer and metal industries, due to limitations in resolution, surface quality, and resulting lack of mechanical strength. Given the potential for functional integration and the complexity of the producible geometries, the ceramic material extrusion additive manufacturing (C-MEAM), and more specifically, the fused deposition of ceramic (FDC) process, is particularly suitable for prototyping structural components. This is especially the case as the conventional fused filament fabrication (FFF) of polymers quickly reaches its limits due to low thermal conductivity and poor heat distortion resistance. Metal additive manufacturing may be unsuitable due to requirements of electrical conductivity and lower corrosion resistance. Such application cases may include electric machines, sensors, power electronics, and chemical or catalytical applications. In contrast, alumina ceramics exhibit excellent thermal conductivity (19–30 W/mK), thermal stability above 1200 °C, and high electrical resistivity (10^15^ Ω·m), making them ideal for such applications [1].

In FDC, the polymers composing the binder are used only as a processing aid for shaping complex parts. The overarching goal is to achieve a homogeneous distribution of the ceramic particles for the sintering process. Inhomogeneous filaments lead to problems during 3D-printing, nonuniform shrinkage, and defects, which adversely affect both the geometric accuracy and the mechanical properties. The current available binder systems used in ceramic 3D-printing are mostly partially debindable in acetone or are not solvent-debindable. These binders can only be post-processed automatically with difficulty. As a solution, the feedstocks system based on a partially water soluble binder and developed in a previous study [2] is investigated. This binder is composed of PVB (polyvinyl butyral), PEG (polyethylene glycol), and additives.

Ceramic additive manufacturing, and specifically, the extrusion-based methods are especially limited by the staircase effect on the surface. This staircase effect is not only visually detrimental but also increases the roughness and can negatively affect the mechanical properties and reliability of 3D-printed components due to stress concentrations [3,4,5]. Ceramic materials are very sensitive to defects, as they do not exhibit plastic deformation like metals [1,6]. Post-machining of sintered ceramic parts is extremely difficult and costly; therefore, green machining is preferred [3].

Since a rough surface is inherent to the FFF process [7,8], several approaches have been studied to improve the surface quality of polymer components. Boparai et al. divide the post-processing methods into two categories: mechanical post-processing methods as in [9], including sanding, machining, abrasive, vibratory, and barrel finishing; and chemical post-processing methods, including painting, coating, heating, and vapor deposition. These methods are further developed in many publications with various in-process or post-processing techniques [10,11,12], like machining [13], thermal tools [14], solvent vapor treatment [15,16,17], chemical dipping [18,19], dip-coating [20], laser polishing [21,22], hot-air polishing [23,24], sandblasting, and vibratory grinding [25], etc. One can also consider other effective process-integrated methods, such as slicing optimization and print-parameter optimization [26,27,28].

However, these results obtained for pure polymers or polymer blends cannot be directly transferred to highly filled polymers (>50 vol% solids). Few publications address the influence of the printing parameters on the surface quality of parts produced by extrusion-based ceramic additive manufacturing [29,30]. The development of additive–subtractive printing strategies is currently developed for pellet-based printers. Furthermore, many of the techniques mentioned above are unsuitable for complex geometries featuring undercuts and channels. Therefore, focus is placed on processes based on dip-coating and solvent-vapor treatment [31,32,33]. These processes either apply material to the surface or partially dissolve the surface binder, allowing viscous flow driven by surface tension [34].

Few publications report the successful use of this binder system for FDC [35] and none demonstrate its surface treatment. To the best of our current knowledge, no published literature exists on dip-coating post-processing of the surfaces of FDC green bodies, giving this topic an exploratory character.

The goal of this study is to achieve structural parts with good surface quality through a dense outer layer, as in ceramic injection molding (CIM), where the parts display high strength despite a core that may contain a higher quantity of porosities. This study is the first to present the effect of dip-coating and aerosol-treatment post-processing on the surface quality of parts produced by FDC. The improvement in the surface quality through these methods may be an economical solution for parts with complex surfaces.

## 2. Material and Methods

### 2.1. Material

The two feedstocks in this study are composed of high-purity alumina powder CT 3000 LS SG, d_50_ = 500 nm, S_BET_ = 7.8 m^2^/g (Almatis GmbH, Frankfurt, Germany), Mowital^®^ PVB resin grade 75H (Kuraray Europe GmbH, Hattersheim, Germany) in the form of fine powder, PEG 6000 (Tecnaro GmbH, Ilsfeld, Germany), and as plasticizer for the PVB resin, triethylenglycol-di-(2-ethylhexanoate) with commercial name OXSOFT 3G8 Cereplas DOA provided by Krahn Ceramics (Krahn Ceramics GmbH, Hamburg, Germany). To the alumina powder, 1500 ppm S30 CR spinel powder (Baikowski, Poisy, France) was added as a sintering additive.

The suspensions are composed of the same base materials, but instead of the plasticizer, they contain KV 9096 dispersant (Zschimmer & Schwarz GmbH und Co., KG, Lahnstein, Germany). Chromium(III) oxide −325 mesh powder from Alfa Aesar (ThermoFischer GmbH, Kandel, Germany) is adjoined to a small extent to add some coloration and differentiate the printed sample with the added dip-coating layer.

### 2.2. Feedstocks

The two most promising feedstocks from the volumetric simplex-centroid mixture design from a previous study were selected [2]: Feedstock 1 and Feedstock 9. Both feedstocks consist of 50 vol.% alumina powder and 50 vol.% binder. Their detailed volumetric composition is listed in Table 1 and the corresponding fabrication procedure is described in a previous publication [35].

### 2.3. Printing

The printing was effectuated on a Prusa i3 MK3 FFF printer (Prusa Research, Praha, Czech Republic) with modified extruder gears to have a better extruder-filament force transfer.

A first printing test on Feedstock 1 was effectuated to assess the range of suitable printing temperature and the effect of air cooling during printing. Two towers were printed with a nozzle temperature between 200 °C and 155 °C.

**Figure 1:**
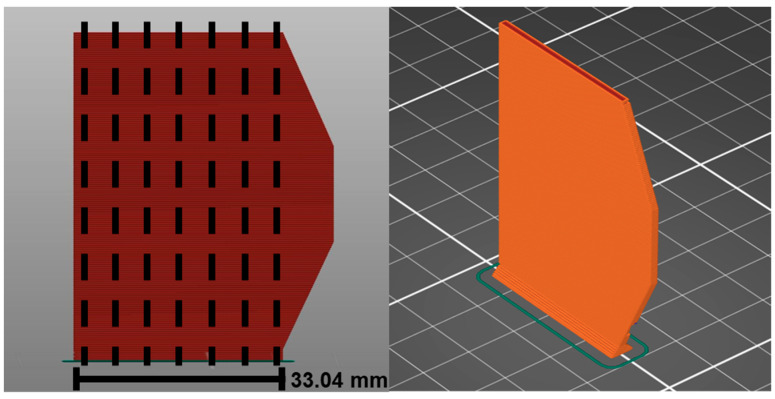
Printed samples face view and position of the cuts (**right**), and 3D schematic (**left**).

To study the mechanical properties in the vertical printing direction (*z*-axis) and to reduce the effect of the edges’ instabilities caused by acceleration or deceleration during printing, vertical parts (cf. Figure 1) with 1.905 mm width and 50.8 mm height were printed with the parameter presented in Table 2. The bending bars were cut in a vertical direction to achieve the appropriate dimensions for the four-point bending test (dotted lines in Figure 1).

### 2.4. Suspensions

For each composition (cf. Table 3), all the constituents were mixed together in a container before the suspensions were deagglomerated on a roller bench for 12 h using 2 mm milling beads.

### 2.5. Dip-Coating

The machine used for the dip-coating of the samples was developed internally at the institute. The machine was designed using FFF and programmed using a micro-controller. The aim was to achieve better control over the process parameters and to enable post-processing of the larger parts. The dip-coating speeds and time were verified and calibrated before use.

**Figure 2:**
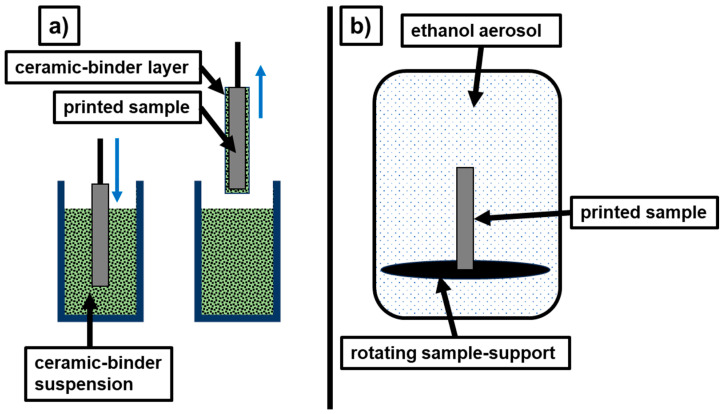
Post-treatment of the printed samples: dip-coating (**a**); ethanol-aerosol based post-processing (**b**).

The dip-coating ceramic suspensions compositions are displayed in Table 3. A schematic of the process is displayed in Figure 2. and the dip-coating parameters that were tested are presented in Table 4.

For the bending bars, the dip-coating suspension nr. 3 (cf. Table 3) was selected consisting of the following: 1.3 wt.% PVB; 0.9 wt.% PEG; 48.6 wt.% alumina powder; 0.8 wt.% additives; and 48.4 wt.% ethanol. During dip-coating, the specimens were immersed in the suspension for 60 s and withdrawn at a speed of 1.66 mm/s. After two hours of drying at 40 °C, the specimens were immersed again using the same parameters and subsequently dried at room temperature.

### 2.6. Aerosol Treatment

The specimens were placed on a sample holder in a vertical position ensuring enough free space around each sample and good contact with the atmosphere. They were treated with ethanol-aerosol created by ultra-sound in a specific post-processing unit (Polysher. Polymaker, Changshu, China) for 30 min. This duration was determined empirically and allows sufficient diffusion of the solvent and softening of the outer layers without inducing significant structural deformation of the sample geometry. Homogeneous distribution of the droplets on the surface of the samples was ensured by the rotating base. The principle of aerosol treatment is shown in Figure 2.

### 2.7. Test Specimens

A total of 32 samples of 50 mm length for both Feedstock 1 and Feedstock 9 were cut from the extruded strand retrieved after the twin-screw homogenization step.

A total of 56 test specimens were successfully cut from the printed parts: 16 samples with Feedstock 1 and 40 samples printed with Feedstock 9.

Only samples produced with Feedstock 9 were post-processed: 12 of them were post-treated by dip-coating and 10 specimens were treated with ethanol aerosol.

Water-debinding was then performed in a water bath at 40 °C for 48 h. Thermal debinding and pre-sintering were performed according to a previously developed program [36] consisting of the following steps: heating at 68 K/h to 160 °C; 2 K/h to 198 °C; 9 K/h to 250 °C; 5 K/h to 282 °C; 9 K/h to 350 °C; 14 K/h to 420 °C; 200 K/h to 800 °C with 3 h holding time; and finally, cooling back to 20 °C at 200 K/h.

The samples were then sintered with a heating rate of 5 K/min from room temperature to 800 °C and 2 K/min until 1600 °C with 120 min holding time at this temperature.

### 2.8. Surface Roughness Measurements

Surface roughness was measured on the green and sintered samples as-processed and surface-treated using a perthometer (Mahr GmbH, Göttingen, Germany). Each sample was measured three times in a row on both sides in order to cover the whole length of the sample. The Rz and Ra values were determined with Mahr Perthometer Concept 6.5 software. Although the surface roughness evaluation is based on a withdrawn standard implemented in the available measurement software, the mathematical definitions of all roughness parameters are displayed below. Owing to the highly periodic nature of the additively manufactured surface, the cutoff wavelength or sampling length was selected according to the spacing of profile elements (RSm, ASME B46.1-2019 [37]), estimated from the layer height and its expected linear shrinkage during sintering. For RSm values ranging from 0.13 mm to 0.4 mm, corresponding to the printing layer height, a cutoff wavelength of λc = 0.8 mm and a corresponding evaluation length *L* of 4 mm is recommended. Additional validation measurements performed using several cutoff wavelengths confirmed that λc = 0.8 mm provides the most representative characterization of the staircase morphology. The selected cutoff wavelength is, therefore, consistent with the standard approach for periodic profile evaluation, even though the resulting non-periodic roughness parameters Ra and Rz may lie outside their conventional validity ranges. The Rz and Ra values were determined using the following formulas:

$$Ra=\;\frac{1}{L}\int_{0}^{L}\left|Z(x)\right|\;dx$$

with *L*, the evaluation length, and *Z*(*x*), the profile deviation from the centerline at position *x*.

$$Rz=\frac{1}{5}({Rz}_{1}+{Rz}_{2}+{Rz}_{3}+{Rz}_{4}+{Rz}_{5})$$

with *Rz*_1_ to *Rz*_5_, the vertical distance between the highest peak and lowest peak of the roughness profile within the overall measuring distance λc.

The results are reported together with the calculated standard deviation of the respective measurements. The standard deviation is calculated with the following formula:

$$\sqrt{\frac{\sum {\left(x-\bar{x}\right)}^{2}}{\left(n-1\right)}}$$

with *x*, the sample value, $\bar{x,}$ the mean value of the samples, and *n*, the number of samples.

### 2.9. Microscopy

Micrographs were taken using an MEF4M light microscope (Leica AG, Vienna, Austria) equipped with an AxioCam camera (Carl Zeiss AG, Oberkochen, Germany).

### 2.10. Bending Test

The strength was investigated in the vertical build direction using a four-points bending test on a Zwick/Roell Z050 (ZwickRoell GmbH & Co., KG, Ulm, Germany) at a crosshead speed of 0.5 mm/min. The 4-point setup has a 20 mm outer span and 10 mm inner span. The results are reported together with the calculated standard deviation of the respective measurements. The standard deviation is calculated with the following formula:

$$\sqrt{\frac{\sum {\left(x-\bar{x}\right)}^{2}}{\left(n-1\right)}}$$

with *x*, the sample value, $\bar{x},$ the mean value of the samples, and *n*, the number of samples.

Because of the brittleness of the printed samples, the pre-loading for these series of measurements was reduced to 25 N and the data acquisition rate increased.

## 3. Results

**Figure 3:**
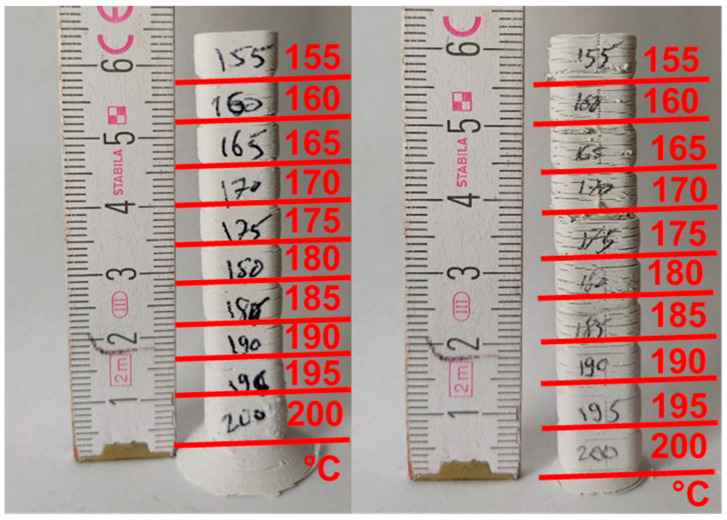
Tours in the green state printed with Feedstock 1 with nozzle temperatures between 200 °C and 155 °C with the cooling fan off (**left**) and on (**right**).

Feedstock 1 demonstrates a broad range of suitable printing temperatures from 155 °C to 200 °C, as can been seen in Figure 3. Feedstock 9 shows similar results. At temperatures of 195 °C and 200 °C, light smoke was observed exiting the nozzle. This indicates that turning the cooling fan on during printing caused delamination and the occurrence of more surface defects (Figure 3 right).

The mechanical test specimens were successfully printed and cut in a vertical direction. The stability during printing was acceptable despite the high aspect ratio of the parts.

**Figure 4:**
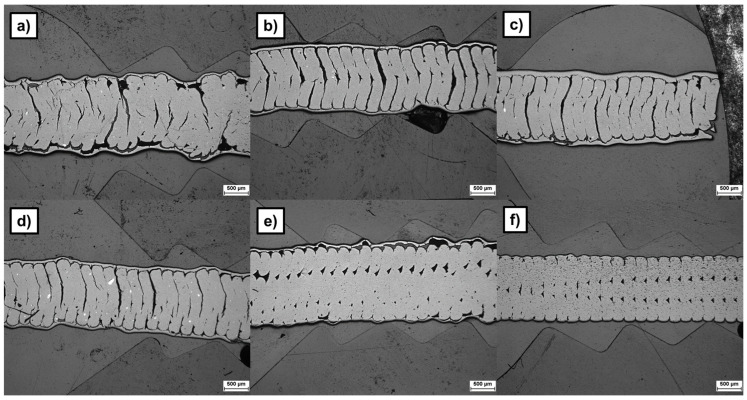
Cross-section of sintered dip-coated and sintered samples with test parameters nr. 1 (**a**); test parameters nr. 2 (**b**); test parameters nr. 3 (**c**); test parameters nr. 4 (**d**); test parameters nr. 5 (**e**); test parameters nr. 6 (**f**) (for respective test parameters cf. Table 4).

The preliminary dip-coating tests were effectuated on samples with lower print quality in order to assess the coating bonding with the irregular surface features. The speeds at which the samples are coated do not present noticeable differences (respectively, Figure 4a at 5 cm/min and Figure 4b at 20 cm/min). Coating the samples twice in a row, as with the test parameters 3 (cf. Figure 4c), provides a thicker and a more homogeneous coating, thus additionally reducing the surface stair-case effect of the printed part. Increasing the dipping time seems to slightly improve the bonding of the layer (cf. Figure 4d,e). The sample processed with test parameters 6, i.e., without dispersant, presents a thin coating compared to the other samples (cf. Figure 4f).

**Figure 5:**
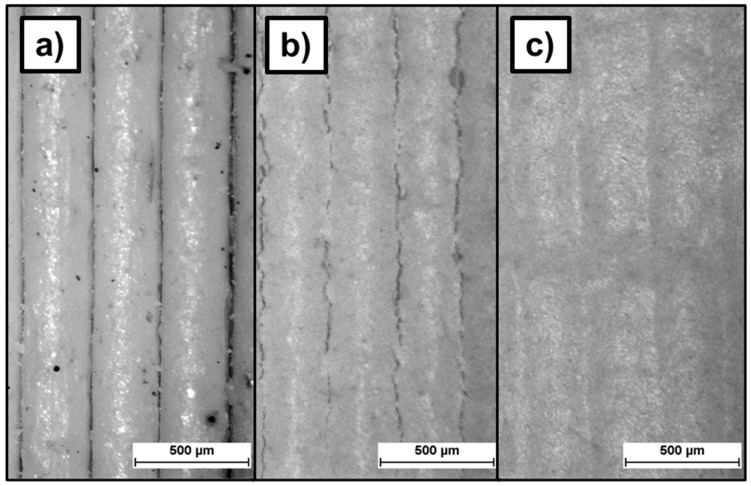
Micrograph of the surface of a green Feedstock 9 test specimen as printed (**a**), after 1 dip-coating (**b**), and after 2 dip-coatings (**c**).

On printed Feedstock 9 bending bars coated with suspension 3 (cf. Table 3) after one dip-coating step, some cracks are visible on the surface caused by the drying of the coating. After the second dip-coating step, these cracks disappear and the surface looks more homogeneous (cf. Figure 5).

The surface roughness of the green bending bars printed with Feedstock 1 and Feedstock 9 displays similar R_z_ values of, respectively, 84.4 ± 18.9 µm, and 90.7 ± 5.3 µm. The R_a_ values are also similar, being, respectively, 19.5 ± 5.0 µm for Feedstock 1 and 21.4 ± 1.3 µm for Feedstock 9. After sintering, these values are reduced by the sintering shrinkage to 73.4 ± 17.4 µm R_z_ and 16.3 ± 4.3 µm R_a_ for Feedstock 1, and 73.5 ± 5.7 µm R_z_ and 16.6 ± 1.3 µm R_a_ for Feedstock 9.

**Figure 6:**
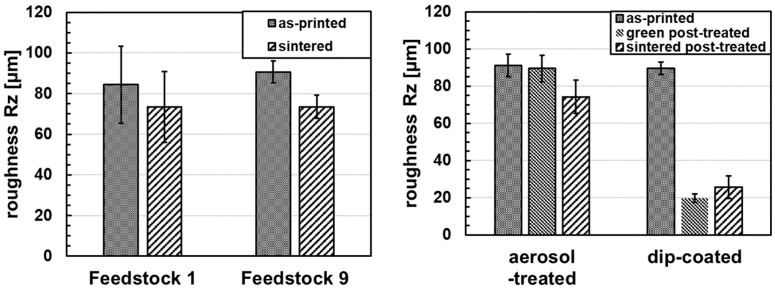
Surface roughness of green and sintered Feedstock 1 and Feedstock 9 bending bars without post-processing (**left**); surface roughness of green and sintered Feedstock 9 bending bars with aerosol and dip-coating post-treatment (**right**).

**Figure 7:**
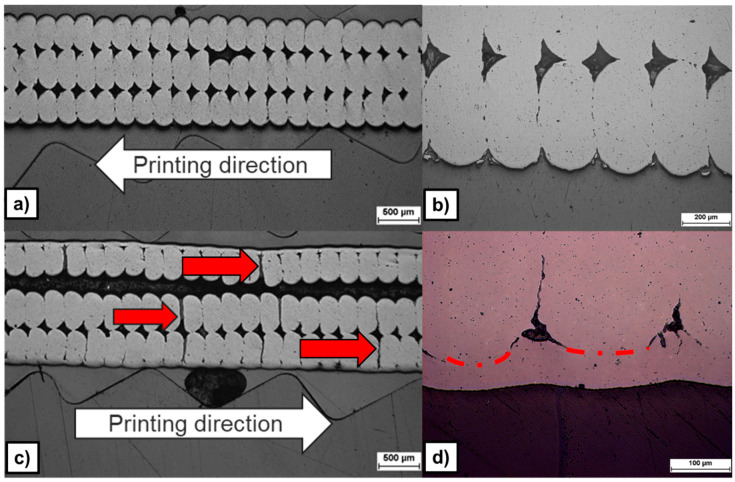
Micrographs of the cross-section of the sintered Feedstock 9 samples after aerosol-treatment (**a**,**b**) and after dip-coating (**c**,**d**).

The aerosol-treated specimens display a surface roughness of 91.2 ± 6.1 µm R_z_ and 21.5 ± 1.3 µm R_a_ before aerosol-treatment, 89.5 ± 7.1 µm R_z_ and 20.9 ± 2.0 µm R_a_ after treatment, and 74.3 ± 9.1 µm R_z_, and 16.3 ± 2.2 µm R_a_ after sintering (cf. Figure 6 right). The aerosol-based post-treatment produced no visible or measurable changes on the surface (cf. Figure 7a,b).

In contrast, dip-coating post-treatment reduced the green-body roughness by 78%, corresponding to a roughness reduction of 65% in the sintered state compared to untreated specimens. The measured roughness was 89.6 ± 3.3 µm R_z_ and 21.2 ± 0.8 µm R_a_ before dip-coating treatment, 19.8 ± 2.3 µm R_z_ and 4.5 ± 0.6 µm R_a_ after treatment in the green state, and 25.5 ± 6.1 µm R_z_ and 5.5 ± 1.4 µm R_a_ in the sintered state (cf. Figure 6 right and Figure 7c,d). At the microscopic scale, the structural bonding between the 3D-printed part and the applied surface coating is steady on the peaks created by the deposited layers. However, the bonding quality remains limited at the macroscopic scale (cf. Figure 7c,d), where pores are present beneath the layer in the interlayer valley regions of the printed structure. These features are present on both Feedstock 1 and 9. Furthermore, the dip-coating step led to delamination between the printed layers (cf. red arrows in Figure 7c).

**Figure 8:**
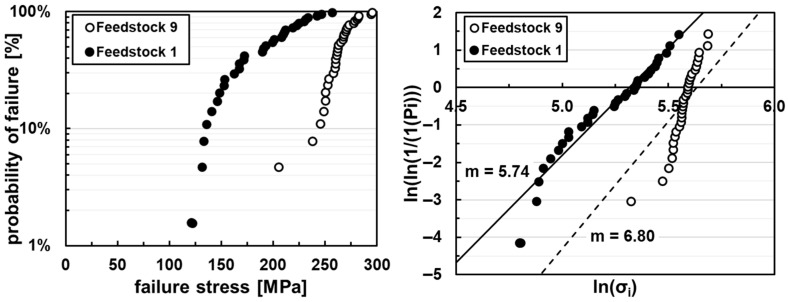
Probability of failure (**right**), and Weibull plot (**left**) for extruded Feedstock 1 and Feedstock 9 sintered bending bars.

The mean stress at failure for the 32 sintered extruded-strand specimens are 188 ± 39 MPa for Feedstock 1 and 259 ± 30 MPa for Feedstock 9. The resulting Weibull modulus is 5.74 for the F1 and 6.80 for the F9 samples (cf. Figure 8).

For the 4-point bending tests of the vertically printed and sintered specimens, several broke before the load was applied. All the dip-coated specimens fractured before the preload of 25 N was attained. For the as-printed Feedstock 1 test specimens, only five measurements could be effectuated successfully because the remaining specimens fractured before preload or during handling, whereas, for Feedstock 9, 13 measurements were performed. For the aerosol-treated F9 specimens, 10 measurement values could be retrieved.

**Figure 9:**
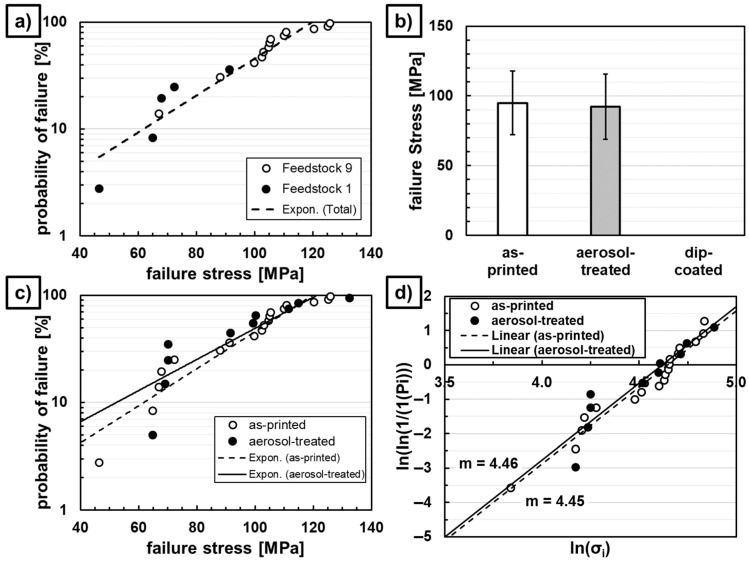
Probability of failure of Feedstock 1 and Feedstock 9 sintered printed bending bars (**a**), and mean failure stress (**b**), probability of failure (**c**), and Weibull plot (**d**) of as-printed, post-treated, and sintered F1 and F9 bending bars.

The samples produced with Feedstock 1 seem to follow the same probability of failure distribution to those of Feedstock 9 (cf. Figure 9a). Therefore, the results of both feedstocks for as-printed specimens are combined in Figure 9b–d for comparison of the mechanical properties to increase representability. The average maximum flexural strength is 95 ± 23 MPa for untreated specimens and 92 ± 23 MPa for aerosol-treated specimens.

## 4. Discussion

The broad temperature range for printing provides parts with good appearance but does not guarantee good interlayer bonding and structural strength. Therefore, the printing temperature for the test specimen was selected just below the obvious decomposition temperature of the feedstock visible from the fumes produced during printing.

The dip-coating preliminary tests show the importance of the dispersant to obtain a stable suspension that slows down the sedimentation, thus allowing a thicker deposited layer. A double dip-coating process and longer dip-coating time lead to more homogeneous surfaces with effective reduction in the staircase effect.

The present study is conceived as an exploratory investigation of process–material relationships in additively manufactured ceramic components, with a particular focus on the influence of surface post-treatments on the staircase effect. Accordingly, surface roughness measurements were conducted with the primary objective of enabling comparative assessments between processing routes rather than providing an exhaustive metrological characterization.

The applied surface roughness methodology is adequate for comparative trend analysis of highly periodic profiles but does not fully comply with conventional filtration requirements for rough surfaces. In particular, the selected cutoff wavelength, although justified for the evaluation of periodic surface morphologies, may place averaged roughness parameters such as Ra and Rz outside their conventional validity ranges. Consequently, the analysis intentionally focuses on a restricted set of parameters. Emphasis is placed on Rz as a pragmatic descriptor of the most severe surface flaws, which are considered most relevant for brittle ceramic materials that are highly sensitive to surface defects and crack initiation. While no direct physical link to fracture mechanics is claimed, the statistical severity of surface defects can be reasonably represented by Rz. Compared to Rmax, Rz exhibits reduced scatter in repeated measurements and thus improved robustness for comparative purposes, whereas Ra averages extreme surface features and provides limited insight into critical flaws.

No formal uncertainty of the measurements data was established, as the surface roughness measurements were intended to provide qualitative and comparative guidance on the effects of post-processing rather than absolute roughness values. A full uncertainty evaluation in accordance with metrological guidelines and advanced statistical analyses were therefore considered disproportionate to the exploratory scope of this work. Furthermore, variations in the number of samples analyzed arose due to process-related damage and limited material availability, such that only intact specimens retrieved from the process chain could be evaluated. While this limits statistical robustness, the observed trends remain consistent across all conditions. These limitations reflect deliberate methodological choices aligned with the exploratory nature of the study and should be considered when interpreting the results.

The surface roughness of both Feedstock 1 and Feedstock 9 displays similar values, but the standard deviation of these roughness values is much higher for Feedstock 1. This could be caused by the higher viscosity of Feedstock 1, causing more irregular flow behavior, thus leading to local over- or under-extrusion and increased variability of the printing. This higher viscosity of Feedstock 1 can be attributed to the increased PVB content, which exhibits a higher molecular weight and a higher intrinsic viscosity than the PEG and the plasticizer.

For the 4-point bending test specimens, the thin printed width of the part with only three adjacent layers was selected to ease assessment of the mechanical improvement caused by the post-treatments by reducing the bulk-to-surface ratio. However, larger part thickness would have improved printing stability and reduced the defects. Furthermore, measuring the bending strength in the vertical direction is challenging due to surface roughness, which can induce stress concentrations at the load application points in contact with the rollers. The limited number of available Feedstock 1 specimens restricts the significance of the results.

The mechanical measurements on the extruded stands give a reference value for the achievable strength of the material without the staircase surface defect. Their mean strength is at least twice the value of the printed specimens. There is a big difference in strength and Weibull modulus between the extruded F1 and F9 specimens. This may be caused by the debinding behavior of the feedstocks. The specimens extruded with Feedstock 1 presented more longitudinal cracks on the failure surface than for Feedstock 9. The debinding has been extensively discussed in a previous publication [2]. The debinding behavior may also differ between extruded and printed samples because of the typical FFF interstrand porosities, thus easing the debinding and possibly reducing the debinding defects for the latter.

The mechanical properties of vertically printed specimens are not directly comparable to those of horizontally printed specimens. The flexural strengths reported by Utsch et al. (mean values between 169 MPa and 200 MPa) for horizontally printed alumina samples tested in a four-point bending configuration fall between the values obtained for vertically printed specimens and those measured for extruded samples [38]. Lithography-based ceramic manufacturing (LCM) of vertically printed specimens yields a flexural strength of 427 MPa and a Weibull modulus of 11.2 [39]. However, this technique is only partially comparable, as its significantly higher process resolution results in fewer and smaller defects.

The brittleness of the dip-coated test specimens caused by their interlayer delamination could be explained by either the contact of the sample with the milling beads at the bottom of the suspension container or by volumetric variations caused by solvent uptake and evaporation. For use of the dip-coating technique in structural applications, further iterations and improvements in both the coating process and the component quality are required. Promising first results were obtained from dip-coating after solvent debinding but are outside the scope of this publication.

The ethanol-aerosol treatment does not improve the surface roughness nor the failure stress or the Weibull modulus. This indicates that such post-treatment does not produce a strengthening effect on the material system. Due to the high amount of ceramic fillers contained and the high molecular weight of the PVB, the softened binder fraction exposed to the aerosol and where the ethanol diffuses is likely to be too viscous to exhibit sufficient flow, which would have led to a rearrangement of the material with the driving forces of gravity, surface energy or capillary effects.

Several alternative solutions could be applied to improve the interlayer bonding and thus the mechanical properties in the vertical direction of the parts and will be investigated in further studies. The addition of a polymer with a low melting temperature could improve the interlayer adhesion [40]. The same effect could be achieved by printing in a heated enclosure. The addition of tackifiers, as described by McNulty et al. in their binder compositions [41], could improve the tackiness of the binder and thus the bonding between the deposited strands, but their effect on the rheology should be carefully monitored. Adding some additives to the ceramic powder could improve the interlayer bonding by reorientation of the grains from the liquid phase sintering and thus improve the overall part strength despite degrading the bulk material mechanical properties.

## 5. Conclusions

Similarly to the weld lines in ceramic injection molding (CIM), the interlayers in FDC represent a weak point in the part, especially in the vertical orientation relative to the printing directions. This vertical direction typically represents the weakest orientation in most additive manufacturing processes. This is particularly the case for FFF. Improving the mechanical properties in this direction would help overcome the strong anisotropy of 3D-printed parts. The surface roughness derived from the staircase effect creates notches and stress concentrations, which can lead to crack initiation. Post-processing of the sintered component is challenging, meaning that surface improvement in the green state is preferable. The objective of this study was to evaluate the compatibility of FDC with the requirements for prototyping structural parts, especially considering the surface quality and the isotropy of the mechanical properties, using alumina as the reference material.

The results demonstrate that:

- Mechanical properties, i.e., failure strength and Weibull modulus, are dependent on the feedstock composition and not only on the constituents (which are the same for both feedstocks).
- Extruded test samples present higher mechanical properties than vertically printed samples.
- The surface roughness can be reduced through dip-coating with a stabilized suspension.
- Stabilizing the suspension gives thicker and more homogeneous coatings.
- Performing two dip-coatings in a row also increases the coating thickness (thus reducing the surface cracks on the coating).
- Aerosol-treatment does not reduce the surface roughness nor improve the mechanical properties of the samples.

However, for the dip-coating treatment, additional work is necessary to ensure that the solution binds in the surface valleys, thus avoiding the formation of pores under the coating. Furthermore, additional care should be taken that the dip-coating step does not compromise the mechanical properties of the parts.

The inherent brittleness of ceramics, especially in combination with the stress concentrations at interlayer interfaces, may represent a potential weak point, although this can be addressed through appropriate structural design. Despite these remaining challenges, the project successfully produced a promising demonstrator component with high surface quality. In the current development state, the process could be applied on aesthetic prototyping parts, where the surface quality is more important than the structural strength, and where the stereolithography-based ceramic manufacturing reaches its dimensional limits due to debinding. For elements requiring tight tolerances, the current process chain is not sufficient without improvements. For parts without a complex surface or inner channels, and where the processing time is less important, subtractive machining of the green part could improve both the dimensional and geometric tolerances and the surface quality.

## Figures and Tables

**Table 1:** Volumetric composition of Feedstock 1 and Feedstock 9.

Feedstock	PVB 75H [vol.%]	PEG 6000 [vol.%]	3G8 [vol.%]	CT 3000 LS SG [vol.%]
F1	16.67	16.67	16.67	50
F9	13.33	23.33	13.33	50

**Table 2:** Bending bars printing parameters in PrusaSlicer 2.7 (Prusa Research, Praha, Czech Republic).

Feedstocks	F1, F9
Nozzle diameter [mm]	0.6
Layer height [mm]	0.3
Print strategy	linear aligned
Printing speed [mm/s]	20
Cooling	OFF

**Table 3:** Composition of the suspensions.

Suspension nr.	1	2	3
PVB 75H [wt.%]	1.4%	1.4%	1.3%
PEG 6000 [wt.%]	0.9%	0.9%	0.9%
CT 3000 LS SG [wt.%]	46.3%	46.6%	48.6%
Chromia powder [wt.%]	0.1%	0.1%	0.1%
Spinell powder [wt.%]	0.1%	0.1%	0.1%
KV 9096 [wt.%]	0.7%	0.0%	0.7%
Ethanol [wt.%]	50.5%	50.9%	48.4%

**Table 4:** Dip-coating parameters.

Test nr.	Suspension	Speed [cm/min]	Dip-Coating Time [s]	Dip-Coating Repetitions	Drying Time [min]
1	1	5	10	1	-
2	1	20	10	1	-
3	1	10	10	2	3
4	1	10	50	1	-
5	1	10	80	1	-
6	2	10	80	1	-
bending-bars	3	10	60	2	120 at 40 °C

## Data Availability

The raw data supporting the conclusions of this article will be made available by the authors on request.
